# Genomic Mechanisms Influencing Outcome in Chronic Myeloid Leukemia

**DOI:** 10.3390/cancers14030620

**Published:** 2022-01-26

**Authors:** Adelina Fernandes, Naranie Shanmuganathan, Susan Branford

**Affiliations:** 1 Department of Genetics and Molecular Pathology, Centre for Cancer Biology, SA Pathology, Adelaide 5000, Australia; adelina.fernandes@sa.gov.au (A.F.); Naranie.Shanmuganathan@sa.gov.au (N.S.); 2 School of Medicine, University of Adelaide, Adelaide 5000, Australia; 3 Precision Medicine Theme, South Australian Health & Medical Research Institute (SAHMRI), Adelaide 5000, Australia; 4 Department of Haematology, Royal Adelaide Hospital and SA Pathology, Adelaide 5000, Australia; 5 School of Pharmacy and Medical Science, University of South Australia, Adelaide 5000, Australia

**Keywords:** BCR::ABL1, chronic myeloid leukemia, next-generation sequencing, TKI resistance

## Abstract

**Simple Summary:**

Chronic myeloid leukemia (CML) is a blood cancer currently well managed with drugs that inhibit the protein responsible for the disease. However, some patients are resistant to these drugs and can progress to fatal phases of CML. This review focuses explicitly on genomic mechanisms that contribute to drug resistance in CML. The ability to predict how patients will respond to treatment at the early stages of the disease is important for selecting optimal therapy and administering more potent drugs before the disease progresses. Currently, only mutations that affect drug binding are included in routine monitoring in drug-resistant patients. This review illustrates other genomic mutations and sequence rearrangements that may impact treatment response and contribute to drug resistance and disease progression. We highlight the potential future role of expanded genomic testing for managing patients with CML.

**Abstract:**

Chronic myeloid leukemia (CML) represents the disease prototype of genetically based diagnosis and management. Tyrosine kinase inhibitors (TKIs), that target the causal BCR::ABL1 fusion protein, exemplify the success of molecularly based therapy. Most patients now have long-term survival; however, TKI resistance is a persistent clinical problem. TKIs are effective in the BCR::ABL1-driven chronic phase of CML but are relatively ineffective for clinically defined advanced phases. Genomic investigation of drug resistance using next-generation sequencing for CML has lagged behind other hematological malignancies. However, emerging data show that genomic abnormalities are likely associated with suboptimal response and drug resistance. This has already been supported by the presence of *BCR::ABL1* kinase domain mutations in drug resistance, which led to the development of more potent TKIs. Next-generation sequencing studies are revealing additional mutations associated with resistance. In this review, we discuss the initiating chromosomal translocation that may not always be a straightforward reciprocal event between chromosomes 9 and 22 but can sometimes be accompanied by sequence deletion, inversion, and rearrangement. These events may biologically reflect a more genomically unstable disease prone to acquire mutations. We also discuss the future role of cancer-related gene mutation analysis for risk stratification in CML.

## 1. Introduction

Chronic myeloid leukemia (CML) is a hematological malignancy that is a prototype for genetically based diagnoses in cancer. This is due to the BCR::ABL1 fusion (HUGO Gene Nomenclature Committee recommended nomenclature [1]), which is an activated tyrosine kinase that forms as a result of a reciprocal t(9;22)(q34;q11) translocation, known as the Philadelphia (Ph) chromosome. The *BCR::ABL1* fusion is the only event necessary to induce CML [2]. Current diagnosis involves detecting *BCR::ABL1* transcripts using qualitative or quantitative reverse transcriptase–polymerase chain reaction (qRT–PCR) or cytogenetic analysis to detect the Ph chromosome [3,4]. Most patients are diagnosed in chronic phase CML, but if left untreated, CML can progress to the more aggressive accelerated phase or terminal blast crisis after 3–5 years [5] which is invariably fatal. The demonstration that BCR::ABL1 induces CML [6] led to the development of tyrosine kinase inhibitors (TKIs) that target and inhibit the fusion oncoprotein. These drugs have revolutionized the treatment landscape of CML. Front-line TKIs were approved for use in the early 2000′s and include (with increasing potency) imatinib, dasatinib, nilotinib, bosutinib, and finally, the third-generation TKI ponatinib, which was developed to target the pan-resistant *BCR::ABL1* kinase domain mutation T315I. A number of novel BCR::ABL1 inhibitors are also in the development pipeline or under investigation in clinical trials [7].

Close monitoring throughout treatment is essential to determine the response. It includes blood counts to assess the hematologic response, cytogenetic assessments of bone marrow to determine the percentage of metaphases that contain the Ph chromosome, and molecular monitoring of peripheral blood *BCR*::*ABL1* transcripts. Most patients rapidly achieve a complete cytogenetic response (no detectable Ph chromosomes in a minimum of 20 metaphases). Molecular monitoring of peripheral blood *BCR*::*ABL1* transcripts is the gold-standard recommended monitoring strategy, as opposed to bone marrow cytogenetics, due to its higher sensitivity and less invasive nature [3,4]. Rapid treatment intervention is mandated for failure to achieve time-dependent treatment milestones.

A major molecular response is defined as *BCR::ABL1* levels of ≤0.1% on the international reporting scale (IS), while a complete cytogenetic response is approximately equivalent to ≤1% *BCR::ABL1* IS [3,4]. While most patients achieve optimal responses when treated with TKIs and remain in the chronic phase of CML long-term, 10–20% develop drug resistance and some even progress to blast crisis CML where the median survival is 12 months [8]. Most patients who achieve optimal responses will remain on TKI therapy indefinitely. Some patients who meet strict treatment response criteria will be able to safely cease therapy and achieve treatment-free remission. This can minimize the toxicity burden of TKI therapy while maintaining the quality of life [3]. However, only 20–30% of all patients will achieve treatment-free remission, and of those who attempt to stop therapy, approximately 50% will experience molecular relapse rapidly [9]. Identifying the factors associated with treatment response and successful treatment-free remission is a long-standing active research area. Despite the dramatic advances in CML, there is currently no reliable diagnostic biomarker that can predict patient outcomes and guide therapy choices for optimal treatment response.

This review explores genomic mechanisms that may influence response to therapy and patient outcome. The detection of these genomic events at different time points, such as diagnosis, drug resistance, and blast crisis, has the potential to aid clinical risk stratification and to identify drug targets. TKI resistance is a significant issue that has been found to have a genomic cause in mutations in the *BCR::ABL1* kinase domain that interfere with drug binding. This finding resulted in the development of TKIs with increased potency that can overcome most of the known imatinib-resistant mutations. *BCR*::*ABL1* mutation analysis is now routine for monitoring patients with treatment failure. However, these mutations are only detected in approximately 50% of resistant patients [10]. Advances in sensitive sequencing technologies, such as next-generation sequencing (NGS), have uncovered further genomic events that could also contribute to resistance. These include mutations in known cancer-related genes and a novel mechanism of genomic heterogeneity that occurs at the time of formation of the Ph chromosome, which we have termed Ph-associated rearrangements [11]. Other genomic mechanisms that have previously been found to contribute to outcomes include the *BCR*::*ABL1* transcript type, derivative chromosome 9 deletions and variant Ph translocations.

## 2. *BCR::ABL1* Kinase Domain Mutations and Their Role in Drug Resistance

While imatinib therapy revolutionized the treatment of CML, reports of imatinib resistance emerged. The best known genomic mechanism contributing to resistance is a mutation within the *BCR::ABL1* kinase domain [12,13,14,15,16,17,18,19], which prompted the development of more potent second and third-generation inhibitors to overcome resistance. These TKIs adhere to the ATP-binding site of BCR::ABL1, inhibiting ATP from binding and preventing tyrosine kinase activation. It thereby inactivates BCR::ABL1 and its subsequent downstream signalling pathways that drive the uncontrolled growth and proliferation of leukemic cells [20]. Thus, kinase domain mutations confer resistance to TKIs by interfering with TKI binding to the ATP-binding site of BCR::ABL1 [12]. More than 70 imatinib-resistant *BCR*::*ABL1* kinase domain mutations have been described thus far. Mutations conferring resistance to second-generation TKIs may also develop, although these mutations are less frequent. Therefore, *BCR*::*ABL1* mutation analysis is recommended for patients with suspected treatment failure as defined by clinical guidelines [3,4] in order to select the most appropriate TKI to rescue response. TKI selection is based on the known TKI sensitivity profile of the various mutations [3,4].

Kinase domain mutations are detected in about half of all patients with acquired resistance [10]. However, they are more frequently identified in either accelerated phase or blast crisis patients, being implicated in 70–80% of resistance cases [21]. It is thought that these mutations arise due to selective pressure of TKI treatment, wherein clones with survival advantages are more likely to proliferate [16,22]. Second and third-generation TKIs, which have increased potency compared with the first-generation TKI imatinib, can overcome resistance conferred by select mutations. However, different mutations confer varying levels of TKI sensitivity, and a substantial proportion of resistant patients treated with more potent inhibitors fail to achieve optimal responses and experience disease progression [23,24,25]. In particular, the T315 residue was predicted to confer resistance [26], and this hypothesis was confirmed in clinical studies where the T315I mutation was associated with a significantly inferior prognosis and overall survival compared with other mutations [12,27,28]. The T315I mutation is particularly resistant to first and second-generation TKIs due to their reliance on a hydrogen bond forming between the threonine at amino acid 315 of *ABL1* and the TKI [29]. Isoleucine at this position disrupts drug binding. Ponatinib, the third-generation TKI, forms a triple bond ethynyl linker with T315, bypassing the reliance on the crucial hydrogen bond [29]. Mutations located in the P-loop of BCR::ABL1 are also associated with poor outcomes and more advanced disease [10,17,19,30,31]. A previous study from our laboratory found that 12 (92%) of 13 patients with P-loop mutations died at a median of 4.5 months (range 0.5–12 months) post detection of the mutation in comparison with 3 (21%) of 14 patients with mutations external to the P-loop [17]. It has also been observed that patients with multiple mutations have a poorer prognosis than those with single mutations [32]. Using NGS, it was found that none of the nine patients that harbored multiple mutations achieved cytogenetic remission, and five of these patients had disease progression [33]. A study from our laboratory observed that multiple low-level mutations conferred lower rates of complete cytogenetic response in comparison with those with a single mutation, even if they had been treated with an appropriate TKI based on the sensitivity profile [34]. Patients with multiple mutations also had a poorer response to ponatinib, which generally suppresses other resistant mutations in addition to T315I [35,36].

With the advent of more sensitive sequencing technologies, the impact of single low-level *BCR*::*ABL1* kinase domain mutations could be analyzed. A study from our laboratory used mass spectrometry to detect low-level dasatinib and nilotinib-resistant mutations in patients who failed imatinib therapy [37]. More sensitive detection identified mutations in 32% of 220 patients, while conventional Sanger sequencing found only 23% with mutations in the same cohort. Mass spectrometry detected an additional 132 mutations in 64 patients, 50 of which were nilotinib or dasatinib resistant. It was found that these low-level resistant mutations predicted lower complete cytogenetic responses when treated with second-generation TKIs, and low-level mutations that were sensitive to a particular second-generation TKI did not expand when that TKI was administered. These findings have been substantiated using NGS [38,39]. NGS detected *BCR*::*ABL1* mutations 6–9 months earlier than Sanger sequencing in patients who failed second-line TKI therapy, and found that low-level mutations can persist even when good outcomes were achieved [40,41]. Mutations associated with resistance detected using NGS were independent predictors of disease progression and loss of cytogenetic response [33]. This is of clinical utility since early detection of mutations can aid treatment decisions and interventions to ensure the appropriate TKI is selected to prevent clonal expansion [39].

There is an ongoing development of TKIs to circumvent TKI resistance and to overcome the toxicity associated with the second and third-generation TKIs. One such TKI is Specifically Targeting the ABL Myristoyl Pocket (STAMP) inhibitor asciminib, which is currently in clinical trial. Asciminib allosterically binds to BCR::ABL1 at the myristoyl-binding pocket, and as a result, the conformation of the ATP-binding domain is altered, and inhibition occurs [7,42]. Thus, asciminib has had success in overcoming resistance due to kinase domain mutations, even in patients with T315I using combination therapy. Significantly, asciminib has reduced toxicity compared with other TKIs due to its high level of target specificity [43,44]. However, in in vitro studies, asciminib-resistant mutations were predicted, especially occurring at the myristoyl-binding pocket that would prevent allosteric inhibitors from binding [7,45]. These mutations have not been identified with clinical resistance to ATP-competitive TKIs.

Nevertheless, in a phase 1 dose-escalation study, myristoyl pocket mutations overlapping the *BCR*::*ABL1* kinase domain have developed in asciminib-treated patients, as detailed in Table 1 [46]. However, current clinical guidelines have not yet included any mutations where asciminib would be contraindicated but will likely be added with the availability of further clinical data [4]. These data indicate the potential relevance of using an ATP-competitive inhibitor in combination with asciminib to reduce the emergence of *BCR::ABL1* mutations. Studies have already found that combination treatment with other TKIs has higher efficacy than single-agent treatment [47,48,49,50]. In vitro studies found that primary CML patient cells treated with asciminib and ponatinib demonstrated decreased BCR::ABL1 activity and colony formation compared with asciminib monotherapy [47]. Mouse models have substantiated these findings with combination therapy showing decreased tumor burden after 21 days compared with a single treatment. Adverse events of ponatinib were also minimized [47]. Similar results have been found in cell lines [50]. As asciminib is a relatively new TKI, further research and clinical trials are required to provide long-term therapeutic data in addition to further insight on the role of *BCR::ABL1* kinase domain mutations for asciminib resistance [44].

To summarize, different mutations confer different degrees of resistance to specific TKIs. Therefore, TKIs must be carefully selected based on their *BCR*::*ABL1* mutation sensitivity profile, as outlined in clinical guidelines [3,4]. The selection of the appropriate TKI is a process that requires careful consideration as inappropriate TKI use can lead to the selection and expansion of a resistant mutation. Hence, early detection of low-level kinase domain mutations can aid this decision.

## 3. Rearrangements Associated with the Formation of the Ph Chromosome

At the formation of the *BCR::ABL1* fusion, genetic variation occurs in the breakpoints involved in the initiating translocation on chromosomes 9 and 22. Furthermore, additional genomic rearrangements accompanying the formation of the *BCR*::*ABL1* gene fusion are also a source of genetic heterogeneity in CML. These can take the form of variant translocations due to the participation of one or more chromosomes in addition to 9 and 22 in the Ph chromosome, which are detectable by bone marrow cytogenetic analysis. In the 1980s, Southern Blot analysis of DNA detected sequence deletions adjacent to the translocation breakpoint that were thought to be of no clinical consequence. In the 1990s, fluorescence in situ hybridization (FISH) techniques detected large sequence deletions at the breakpoint on the derivative chromosome 9 that spanned several hundred kilobases. The deletion status was found to be an independent predictor of poor patient outcomes. With the advent of advanced sequencing technologies in the 2000s, the genomic complexity accompanying the formation of the Ph chromosome became evident with the detection of novel gene fusions and complex rearrangements that included sequence inversion and fragmentation. As technology has advanced, the resolution of the detection of complex genomic events has been enhanced. Cytogenetic and FISH analysis are still used in the management of CML, particularly at diagnosis and resistance assessment, since molecular analysis has largely replaced these techniques for long-term monitoring of treatment response. In this section, we discuss the clinical impact of additional rearrangements and how NGS has significantly expanded our understanding of the complex nature of these rearrangements.

### 3.1. The Influence of BCR::ABL1 Transcript Type and Treatment Response

The most common breakpoint in CML occurs in the 5.8kb major breakpoint region of *BCR* (M-BCR) and a 140kb region between exons 1b and 2 of *ABL1*. This event results in a 210 kDa BCR::ABL1 protein and common *BCR*::*ABL1* transcripts where either *BCR* exon 13 or 14 are fused to *ABL1* exon 2. These transcripts are termed e13a2 (previously known as b2a2) and e14a2 (previously known as b3a2), and a proportion of patients express both transcripts due to alternative splicing [52]. These common *BCR*::*ABL1* transcripts occur in >98% of CML patients [53]. The e14a2 transcript is more common than e13a2 (62.1% [including patients expressing both transcripts] versus 37.9%). Coexpression of both transcripts ranged between 1.1% and 26.9% [53].

Atypical *BCR::ABL1* transcripts are rare and occur in 1.93% of patients [53]. These include the e1a2 transcript that occurs in 0.91% of patients. This is formed as a result of the breakpoint lying in a 72kb region of intron 1 of *BCR*, known as the minor breakpoint region (m-BCR) and generates a 190 kDa BCR::ABL1 protein. A third transcript type generates a p230 kDa BCR::ABL1 protein and an e19a2 *BCR*::*ABL1* transcript. The breakpoint usually occurs within *BCR* intron 19 in the μ-BCR region [54] and are rarer, with incidences of the e19a2 transcript found to be 0.31% [53]. Other atypical transcripts include e13a3, e14a3, e1a3, e6a2, e8a2, e1a1, e8a1, e8a3, e15a2, and e23a1 [53].

There are conflicting findings as to whether this genetic variation in transcript type impacts treatment outcome [55,56,57]. Studies in the pre imatinib era were conflicting, and therefore this could not be used as a reliable prognostic factor. Higher rates of major cytogenetic response (≤35% Ph chromosome levels) were reported in patients with e14a2 and a trend for poorer outcome with e13a2, while several studies found no significant difference between the two transcript types [58,59,60] as reviewed by Molica et al. and Sharma et al. [55,56]. In the TKI era, studies have found that patients with e14a2 had a better imatinib response than those with e13a2 [61,62,63], whereas some studies found that e13a2 had higher rates of major cytogenetic response and ten-year overall survival [55,64]. However, other studies found no difference in cytogenetic and molecular response between transcript types [61,65]. We have not found differences in response according to transcript type for most responses, except for a higher rate of deep molecular response for patients with the e14a2 transcript [57]. Second and third-generation TKIs have been reported to produce similar response rates for both transcript types, and responses may be superior in patients with e13a2 compared with responses for imatinib-treated patients [62,66]. However, as these findings have not yet been validated on larger prospective cohort studies, current treatment guidelines do not include transcript type for treatment decisions. Furthermore, careful monitoring of patients and rapid treatment intervention for failure to achieve milestone molecular responses can overcome any differences in outcome [3,4].

The *BCR*::*ABL1* transcript type has also been found to influence the success of treatment-free remission. Studies reported that patients with the e14a2 transcript had a higher probability of achieving sustained MR4.5 (*BCR::ABL1* ≤ 0.0032% IS), which is a deep molecular response and a prerequisite for stopping therapy in an attempt to achieve treatment-free remission [66,67]. This finding is substantiated by studies showing that patients with e14a2 were more likely to maintain treatment-free remission [68,69,70]. Work from our laboratory found that 67% of patients with the e14a2 transcript maintained treatment-free remission at 12 months after cessation compared with 40% with the e13a2 transcript [68]. Therefore, determination of transcript type could be of clinical utility in determining the probability of treatment-free remission. The reason for this potential difference in treatment outcome due to transcript type remains unknown, although the e14a2 transcript is more immunogenic and could elicit a T-cell response that aids the elimination of *BCR*::*ABL1* transcripts [71,72]. Furthermore, Lucas et al. found elevated levels of phosphorylated CrKL in patients with the e13a2 transcript, which indicates higher tyrosine kinase activity of BCR::ABL1 [63]. However, the molecular basis for this activity is still poorly understood [63]. The impact of atypical transcripts on outcome remains unclear due to the rarity of their occurrence. Patients with atypical transcripts are currently not recommended for attempting treatment-free remission due to lack of standardized monitoring; however, preliminary data have suggested that this may become a possibility conditional to more effective molecular monitoring for these patients [73,74]. In summary, the impact of transcript type on patient outcomes is an area of debate in the pre and post-TKI era; however, there has been no observed impact on responses to second and third-generation TKIs. Studies have found that transcript type impacts treatment-free remission and therefore could be a factor in risk stratification of patients attempting treatment-free remission.

### 3.2. Variant Translocations

**Figure 1:**
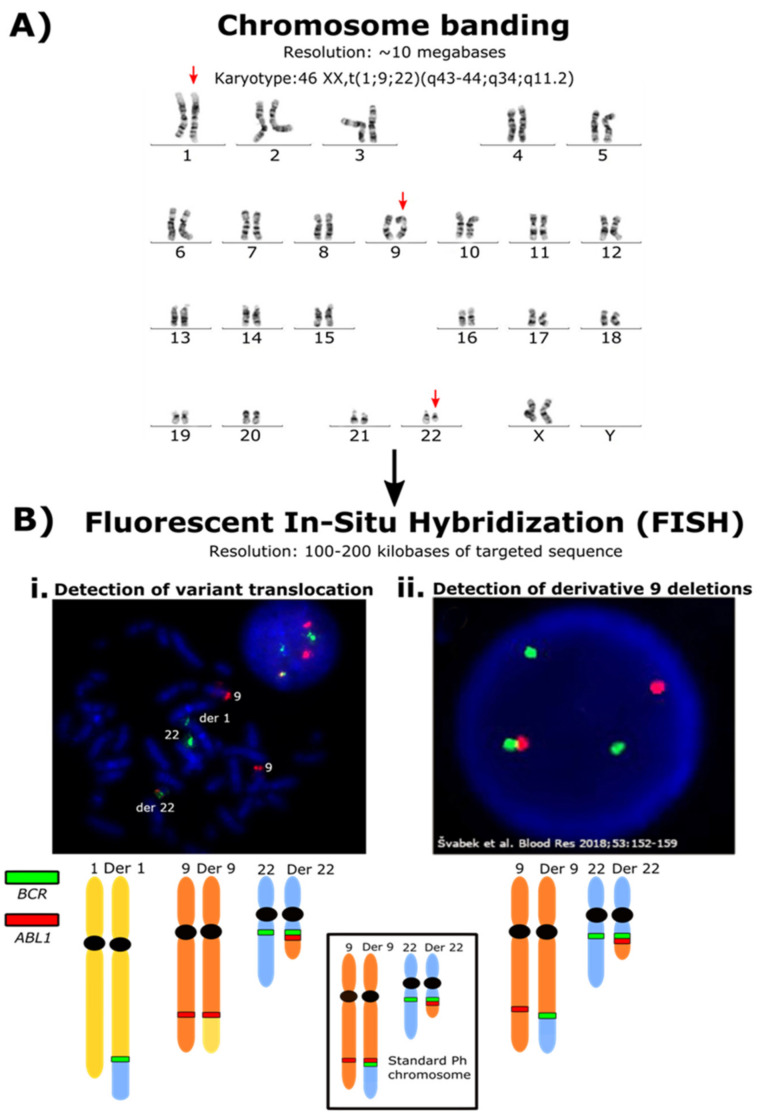
Increasing resolution of techniques for detecting rearrangements associated with the formation of the Ph chromosome. (**A**) Karyotype of a patient with a 3-way variant Ph chromosome. Using chromosome banding, this large-scale chromosomal rearrangement was detectable. The arrows indicate the chromosomes involved in the translocation. The Ph chromosome is the visibly shortened chromosome 22, where the end of the q arm was translocated to chromosome 1. This fragment usually translocates to the derivative chromosome 9. The end of the q arm of chromosome 1 was translocated to the derivative chromosome 9. The resolution of chromosome banding is limited, and the specific genes involved in these rearrangements are undetectable. (**B**) **i.** FISH analysis using a BCR/ABL dual color dual fusion probe and metaphase spread confirmed the presence of the *BCR::ABL1* fusion signal (red and green signal on the derivative (der) 22) but the absence of the reciprocal *ABL1::BCR* fusion signal on the der 9. The *BCR* gene locus (green signal) was translocated to the der 1. The FISH analysis is not able to define the exact location of the fused sequence of chromosome 22 within the der 1 or any genes that may be associated with a gene fusion involving *BCR*. **ii.** Derivative 9 deletions are also detectable by FISH analysis. The figure represents an *ABL1* deletion of an interphase nucleus derived from Figure 2D from Švabek et al. [107]. These are below the size resolution for detection using chromosome banding. The deletion is indicated by the lack of a red *ABL1* signal joined to a green *BCR* signal. The FISH analysis will only detect the regions targeted by the fluorescent probes and cannot identify novel fusion gene partners. The image is reproduced through the terms of the Creative Commons Attribution 4.0 International License (http://creativecommons.org/licenses/by/4.0/ accessed on 2 January 2022).

In most patients, the *BCR*::*ABL1* fusion is formed when the terminal sequence of the q arm of chromosome 9 is swapped with the terminal sequence of the q arm of chromosome 22. The genomic breakpoints usually occur within the *BCR* gene on chromosome 22 and the *ABL1* gene on chromosome 9. Beginning with the initiating translocation event, genetic heterogeneity in CML is detectable cytogenetically in some patients by observing variant translocations (Figure 1). Genetic events involving other chromosomes in addition to 9 and 22 in the formation of the *BCR::ABL1* fusion are termed variant translocations [75]. These occur in 5–10% of newly diagnosed CML patients [76,77]. It has been debated whether variant translocations are formed simultaneously with *BCR::ABL1* formation in a single genomic event or in a two-step process wherein *BCR::ABL1* is formed first, and other translocations occur subsequently [78]. Studies showed evidence for a one- or two-step mechanism of variant translocation formation in CML patients using FISH [76,77,79]. A study by Calabrese et al. found the third breakpoint on the derivative chromosome 9 that was separate from the one involved in the formation of the Ph chromosome [80]. Material from chromosome 22 translocated into a third partner chromosome, including a sequence from the derivative 9 breakpoint, suggesting a second translocation [80]. The most common partner chromosome involved in variant translocations is chromosome 17; however, chromosomes 3, 6, 7, 8, 9, 11, 12, 16, and 19 have also been reported [75,76,77,81,82,83].

It has also been debated whether patients with variant translocations have inferior outcomes. Deletions on the derivative chromosome 9 adjacent to *ABL1* were found more frequently in patients with a variant translocation and occurred in 40.4% of these patients compared with 12% in patients with a classical translocation [76,82,84,85]. Derivative 9 deletions were predictive of adverse outcomes (explored subsequently in this review), suggesting that variant translocations would confer a poorer prognosis. In the pre-TKI era, patients with variant translocations did indeed have a poorer outcome and shorter survival in some studies than those with a classical translocation [85,86,87]. However, studies in multiple large international cohorts of imatinib-treated patients found no significant difference in molecular outcomes, cytogenetic response or overall survival between patients with variant or classical translocations [83,88,89,90,91]. The mechanism of variant translocation formation was also irrelevant to predict imatinib response [92]. In contrast, Stagno et al. showed that patients with a variant translocation had poorer cytogenetic and molecular responses at 6–18 months from starting imatinib or nilotinib compared with patients without variant translocations [93]. However, these observations were derived from only ten cases, preventing definitive conclusions. Gorusu et al. further compared patients with a derivative 9 deletion in addition to a variant translocation and found that these patients had a poorer cytogenetic response to imatinib than those with only a variant translocation [76].

Another form of genetic heterogeneity in the formation of *BCR::ABL1* is Ph-negative CML. The Ph chromosome is not cytogenetically detectable in these patients due to rearrangements such as insertions of *ABL1* into *BCR* or subsequent translocations that restore the Ph chromosome to normal morphology [94]. In cases of Ph-negative CML, the *BCR::ABL1* fusion is still detectable using methods such as FISH [75,95], and *BCR::ABL1* transcripts are detectable. Ph-negative CML is rare, occurs in about 1–2% of patients, and has no prognostic impact [96,97,98]. To summarize, although early research found that variant translocations conferred poorer outcomes in the pre-TKI era, with the introduction of TKIs and good clinical guidance for treatment intervention, variant translocations no longer have prognostic implications [99].

### 3.3. Derivative Chromosome 9 Deletions

Further genetic heterogeneity in CML is evident by deletions on the derivative chromosome 9 adjacent to the translocated *ABL1* and *BCR* genes [84]. These were first detectable using FISH analysis (Figure 1). The deletions spanned the translocation breakpoints and involved several megabases of chromosome 9 and 22 sequences. They are believed to occur during the formation of the Ph chromosome rather than disease progression and were detected in 9–15% of CML patients [100,101]. They were consistently associated with rapid disease transformation and shorter survival and were a more powerful predictor of outcomes than clinical scoring systems when first identified [84]. The size of the deletion was a factor for outcome, but some technologies could not adequately distinguish size. Underpinning earlier research, array-comparative genomic hybridization could also detect these deletions. Five of the 49 patients tested in one study had copy number variations that were cryptic deletions on the derivative chromosome 9 [102]. All 49 patients had no cytogenetically detectable chromosomal anomalies other than the Ph chromosome. In the pre-TKI era, studies found that patients with a derivative 9 deletion had significantly shorter median overall survival and faster time to disease progression [82,84,101,103]. This difference has been largely overcome by introducing TKIs, and most studies did not find a significant difference in outcomes or overall survival between patients with or without derivative 9 deletions when treated with imatinib and second-generation TKIs [101,104,105]. Contradictorily, a study by Huntly et al. found that imatinib-treated patients with derivative 9 deletions had lower hematological response rates and major cytogenetic response and faster time to disease progression than those without these deletions [100]. The relevance of derivative 9 deletions was still debated in the early years after the introduction of TKIs, and the 2006 European LeukemiaNet guidelines for the management of CML suggested that derivative 9 deletions were candidate prognostic factors [106]. However, the current consensus, supported by the majority of studies, is that derivative 9 deletions are of no prognostic significance [99].

### 3.4. Ph-Associated Rearrangements

**Figure 2:**
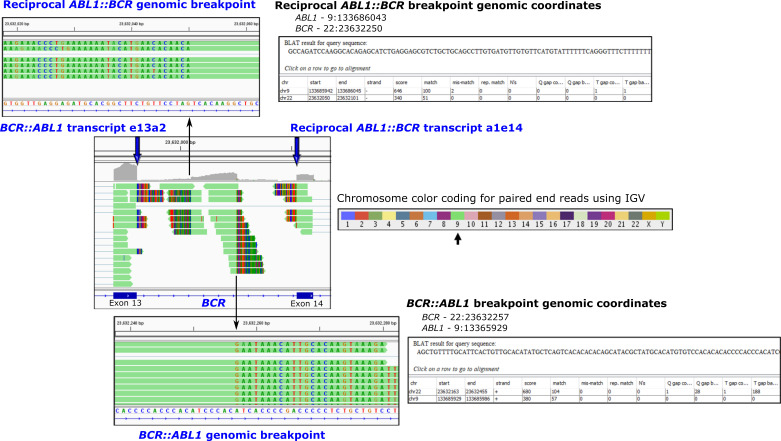
Fusion transcripts and the corresponding genomic breakpoints at base-pair resolution are detectable using next-generation sequencing. Shown is a segment of the *BCR* gene on chromosome 22 for a patient with the *BCR::ABL1* fusion gene and CML where paired-end sequencing reads are visualised using the Integrative Genomics Viewer (IGV). RNA-seq using total RNA was performed [11]. Bioinformatic tools identify the rearrangements, and IGV allows a composite view of all fusions associated with the translocation between chromosomes 9 and 22. The multicoloured regions (chimeric reads) indicate that the sequence is derived from another location in the genome. The color-coded reads indicate that the sequence is derived from chromosome 9. Zooming into the *BCR* intronic region reveals the fusion breakpoints at base-pair resolution. Alignment of the chimeric reads using the BLAT function indicates the genomic coordinates of the partner read. Fusions include the primary disease-causing *BCR::ABL1* oncogenic fusion transcript (*BCR* exon 13 fused to *ABL1* exon 2) and the corresponding intronic *BCR::ABL1* genomic fusion. These rearrangements are located on the Philadelphia chromosome. The reciprocal rearrangements located on the derivative chromosome 9 are also detectable in the IGV composite view: the reciprocal *ABL1::BCR* fusion transcript (*ABL1* exon 1 fused to *BCR* exon 14) and the reciprocal *ABL1::BCR* genomic fusion. Genome build hg19.

We have recently identified a novel mechanism of genetic heterogeneity in the formation of the Ph chromosome using higher resolution NGS analysis that we have termed Ph-associated rearrangements [11]. Typically, the translocation generates the *BCR*::*ABL1* fusion, and in about 50% of patients, the reciprocal *ABL1*::*BCR* transcript is also generated (Figure 2). In patients with Ph-associated rearrangements, the *BCR*::*ABL1* fusions were formed as usual, but the *ABL1*::*BCR* fusions were absent. Instead, *BCR* or *ABL1* were fused to other genes on chromosome 9 or 22, or genes adjacent to *BCR* or *ABL1* formed gene fusions. Sequence deletions and inversions were also evident. In some cases, fusion involving genes on other chromosomes were detected and were consistent with variant translocations identified by cytogenetic analysis. These rearrangements were detectable at diagnosis and did not emerge at blast crisis, indicating they likely occurred at the time of formation of the Ph chromosome. Importantly, Ph-associated rearrangements were associated with a poor outcome where 33% of patients who progressed to blast crisis on first-line TKI treatment had a Ph-associated rearrangement compared with 11% of patients who achieved an optimal response [11]. Outcome prediction may improve with further classification of deletions and complex chromosomal rearrangements associated with the initiating translocation using new technology, revealing further genomic complexity in some patients. Future studies will determine whether Ph-associated rearrangements are prognostic markers that could guide up-front treatment decisions.

Additional rearrangements have been reported with fusion transcript generating translocations in other malignancies, and some may indicate an inferior patient outcome. In acute leukemia, rearrangements such as submicroscopic deletions and inversions have been associated with known and novel fusions, including *KMT2A* (*MLL*), *ETV6::RUNX1*, *CBFB::MYH11* and *RUNX1::RUNX1T1* [108,109,110,111]. In patients with AML, the *RUNX1::RUNX1T1* fusion is generated by a translocation between chromosomes 8 and 21 and confers a favorable prognosis. However, the prognosis can be altered when the translocation involves complex rearrangements with additional chromosome involvement or cryptic rearrangements where the sequence is inverted [112]. These events may indicate larger-scale genomic instability associated with a poorer outcome.

Childhood *KMT2A* rearranged acute lymphoblastic leukemia (ALL) is associated with a poor prognosis, and there are multiple different *KMT2A* fusion partners. More than half of the rearrangements are complex, involve three or more chromosomes, and are accompanied by large deletions or inversions adjacent to the breakpoints [113]. These are similar to the Ph-associated rearrangements that we have reported in CML [11]. Interestingly, one study reported a relatively favorable outcome for *KMT2A*-rearranged childhood ALL for a subset of patients where the reciprocal rearrangements were detected [114], analogous to the reciprocal *ABL1*::*BCR* fusions identified in CML patients. The ALL patients that lacked the reciprocal fusion exhibited complex translocations or carried a single *KMT2A* rearrangement [114]. We found that the reciprocal *ABL1*::*BCR* fusion was not detected in patients with Ph-associated rearrangements [11]. The Ph-associated rearrangements were an indicator of more complex rearrangements and were associated with a poor outcome [11]. They could be a marker of a genetically unstable disease prone to acquire additional disease transforming mutations. Whether ALL patients with *KMT2A*-rearrangements lacking reciprocal fusion are also markers of a genetically more unstable disease is unknown.

Similarly, complex gene fusion generating rearrangements have been reported in solid tumors, such as *EWSR1::ERG* and some *EWSR1::FL11* fusions in Ewing sarcoma, wherein the rearrangements involve inversions and other complex rearrangements rather than simple reciprocal translocations [115]. These complex rearrangements involved multiple genes on different chromosomes rearranged in closed loops. This type of rearrangement is known as chromoplexy, defined as a series of interdependent rearrangements among multiple chromosomes [116,117]. Notably, the chromoplectic rearrangements were a marker of aggressive disease, and patients were more likely to relapse in comparison with patients with fusions that were formed by simple rearrangements.

**Figure 3:**
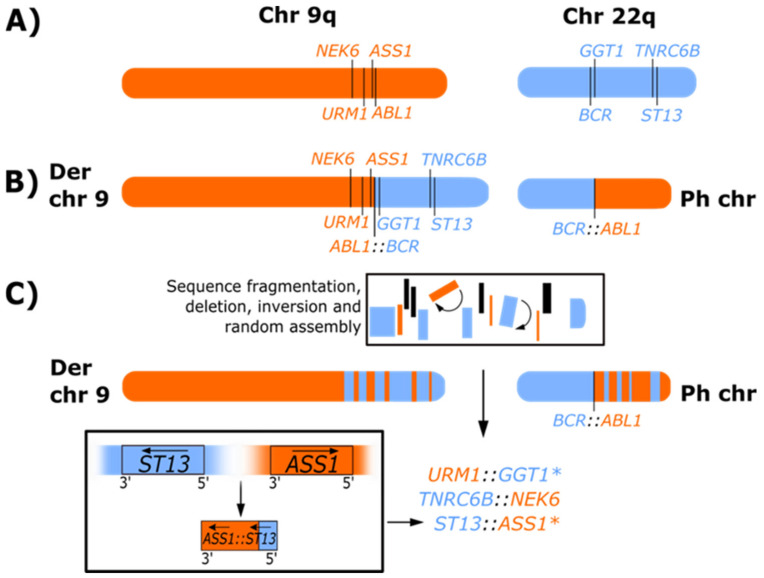
Representation of Ph-associated rearrangements formed as a result of chromoplexy-like events. (**A**) Representation of the q arms of normal chromosomes (chr) 9 and 22. (**B**) Classical Ph chromosome and derivative (Der) chromosome 9 formed by a reciprocal translocation in CML. (**C**) Representation of Ph-associated rearrangements observed for patient 24 of our whole exome sequencing, RNA-seq and copy number variation analysis [11] where multiple novel gene fusions were detected. The location and orientation of the gene fusions suggest that segments of chromosome 9 (orange) and chromosome 22 (blue) fragmented during the formation of the Ph chromosome, which resulted in the loss of genetic material (indicated in black), inversion (indicated by arc arrows and denoted with (*), and random reassembly of sequence that generated novel fusions. Using *ST13:ASS1* as a model, the fusion was formed following the deletion of sequence adjacent to *ST13* on chromosome 22 and *ASS1* on chromosome 9, and complex rearrangement involving inversion (denoted with (*) and reassembly of sequence that brought the genes into the same transcriptional orientation. The arrows in the lower box indicate the original transcriptional direction of the genes on their respective chromosomes and the transcriptional direction after deletion, inversion, and sequence reassembly. Consistent with other patients with Ph-associated rearrangements, the reciprocal *ABL1::BCR* transcript on derivative 9 was not detected due to disruption and deletion of adjacent sequence. The complete sequence of events is not fully resolved, but the positioning of the fusion partners of the novel fusions suggests that sequence fragmentation may have occurred on both the derivative 9 and the Ph chromosome, and the novel fusions may be located on derivative 9 or the Ph chromosome.

Chromoplexy was first discovered in patients with prostate cancer involving fusions such as *TMPRSS2::ERG* [117]. These can involve complex chains of rearrangements and sequence deletion where DNA sequence is shuffled after breakage and re-ligation of genes or DNA segments in a random configuration. We identified similar rearrangements in some patients with CML, although only two chromosomes were involved [11]. Figure 3 shows an example of chromoplexy-like rearrangements in a CML patient at diagnosis [11]. Whole exome sequencing, copy number variation analysis and RNA-seq identified multiple deletions on chromosomes 9 and 22 in the region of the *BCR* and *ABL1* genes, and multiple novel gene fusions in addition to the *BCR*::*ABL1* fusion. Some of the novel fusions involved inversions that brought genes into the same transcriptional orientation. Furthermore, the unusual positioning of the fusion partners as either the 5′ or 3′ gene, relative to their original locations on chromosome 9 or 22, indicates that sequence fragmentation and random reassembly of genes likely occurred. The incidence of complex rearrangements in multiple cancers and a noticeable impact on outcome in some cases align with our finding of a poorer outcome for CML patients with Ph-associated rearrangements with the chromoplexy-like mechanism of action.

To summarize, genomic events similar to Ph-associated rearrangements have been found in other hematological and solid tumor malignancies. These may adversely impact outcomes, which supports our findings in CML. Further studies focusing on the impact of the Ph-associated rearrangements on treatment outcome could potentially enhance clinical risk prediction for patients with CML and aid the selection of first-line TKI treatment.

## 4. Role of Potentially Pathogenic Variants in Cancer Genes, Including Fusions and Deletions

Our understanding of the mechanisms of drug resistance and treatment failure beyond *BCR*::*ABL1* mutations has recently improved. NGS studies have confirmed the role of mutations in cancer-related genes [11,118,119,120,121,122]. Early studies first observed that *RUNX1,* a regulator of hematopoietic cell differentiation [123], was mutated in some patients in blast crisis CML [124]. Studies in the last decade have confirmed the role of *RUNX1* mutations, including gene fusions, in the transformation to blast crisis [11,118,119,121,125,126]. Downregulation of DNA repair genes has been demonstrated in the *RUNX1*-mutated blast crisis [127]. Roche-Lestienne et al. conducted an initial study using genomic DNA of four cancer genes that had been commonly reported in *BCR::ABL1* negative hematological cancers: *TET2, IDH1, IDH2,* and *ASXL1*. This study found no *IDH1* or *IDH2* mutations and *TET2* and *ASXL1* in only a few patients [128]. A more sensitive and more extensive analysis was conducted by our laboratory using integrative genomic analysis [11]. We found that cancer gene mutations were detectable at diagnosis in 50% of the 46 patients treated with first-line TKIs; 70% of 27 patients who did not achieve a significant molecular response had a cancer gene mutation in comparison with 21% of 19 patients who did achieve this response [11]. Furthermore, cancer gene mutations were found in all patients diagnosed in blast crisis. We found *ASXL1* to be the most frequently mutated gene. These findings have been validated by other studies, which found that cancer gene mutations were more frequent at diagnosis in patients who progressed to blast crisis and were therefore associated with inferior outcomes [119,126]. *ASXL1* was also found to be one of the most frequently mutated genes [119,120,126]. There were discrepancies in the frequency of mutations detected at diagnosis in chronic phase patients between studies with frequencies ranging from 29% to 50% [129].

A study by Wu et al. observed an increased frequency of *ASXL1* associated with *BCR::ABL1* kinase domain mutations [120]. This study also investigated the frequency of mutations in patients with TKI intolerance. They reported that mutations in *CUX1, KIT,* and *GATA2* may play a role in TKI intolerance as these mutations were found at higher rates in intolerant patients than TKI resistant patients. The authors found that these genes were myelodysplastic syndrome-related genes and mutations affected hematopoiesis, especially in relation to cytopenias, which are associated with intolerance [120]. The most commonly mutated cancer genes are *ASXL1, RUNX1, IKZF1, BCORL1, KMT2D, DNMT3A, JAK2, TP53* and *TET2*. *IDH1* and *IDH2* were rare, consistent with earlier studies [130].

Recent research by Zhang et al. and Xue et al. have confirmed that *ASXL1* is the most frequently mutated gene in CML [131,132]. Zhang et al. found that 87% of the 169 CML patients in the chronic phase or accelerated phase treated with a third-generation TKI for drug resistance harbored one or more cancer gene mutations [131]. Mutated *ASXL1* occurred in 69% of patients. The poorest response and outcome to third-generation TKIs occurred for those patients with three or more cancer gene mutations. Furthermore, cancer gene mutations and additional chromosomal abnormalities were independently associated with progression-free survival. Certain additional chromosomal abnormalities acquired during therapy are currently considered a criterion for treatment failure [3], and future guidelines may incorporate cancer gene mutations into the treatment failure algorithm. Importantly, Zhang et al. identified patient risk groups (favorable, intermediate, and high) based on the *ASXL1* mutation status and variant allele frequency, which demonstrates the potential power of genomic testing for risk stratification. Complementary to these findings, Xue et al. [132] found that cancer gene mutations were detectable in all disease phases, and the highest frequency occurred in accelerated and blast phase at ≥85%. *ASXL1* mutations were the most common in each disease phase. Furthermore, in vitro modelling of an *ASXL1* mutation in *BCR*::*ABL1*-transformed cells found that this mutation conferred a significant degree of resistance to first and second-generation TKIs. Further studies are warranted to assess the TKI sensitivity of other common mutations reported in CML. These future findings could influence treatment decisions.

There are currently no clinical guidelines for diagnostic monitoring of cancer gene mutations [3] or guidance on therapeutic intervention if a pathogenic mutation is identified [4]. The U.S.-based National Comprehensive Cancer Network Clinical Practice Guidelines in Oncology for CML suggest that NGS using a myeloid mutation panel should be considered for patients who present in advanced disease phases or have disease progression to advanced phases with no identifiable *BCR::ABL1* kinase domain mutation [4]. However, lymphoid gene mutations may also be relevant for lymphoid phenotype blast crisis CML and *BCR*::*ABL1* mutations frequently co-occur with mutated cancer genes [11,125]. In blast crisis, 85% to 100% of patients with *BCR*::*ABL1* kinase domain mutations also had cancer gene mutations, including gene fusions and deletions [11,125,133]. Known and novel gene fusions in CML have now been described in multiple studies of CML resistance [11,121,126]. Furthermore, deletions involving the *IKZF1* gene are among the most frequently detected in lymphoid blast crises [11,125,133]. Therefore, restricting mutation analysis to single nucleotide variants and small insertions and deletions in myeloid genes may fail to detect a substantial proportion of relevant mutation types.

There is a growing body of evidence indicating that the detection of cancer gene mutations has the potential to be utilized for risk stratification and could guide treatment decisions [130]. Further validation of NGS results on larger unselected cohorts will provide sound evidence for future clinical practice guidelines for managing cancer gene mutation-driven resistance in CML. An area of research that remains incomplete is the functional impact of the various cancer gene mutations in a CML context. Some research has already been conducted on mutant *ASXL1* as mentioned above [132], as well as mutated *RUNX1* and *UBE2A* that have been recurrently mutated in CML. Studies using Ba/F3 transfected with *BCR::ABL1* and the K562 CML cell line found that *UBE2A* mutations contributed to the downregulation of myeloid differentiation pathways [122]. *RUNX1* mutations contributed to the downregulation of DNA repair machinery and promoted genomic instability [127]. This indicates the potential for further functional work on other cancer genes in CML to direct future therapeutic targets independent of *BCR::ABL1*. Hence, cancer gene mutations could potentially assist in guiding a more personalized medicine approach to treating CML.

## 5. Conclusions

It is abundantly evident that despite the consistent genetic signature of *BCR::ABL1*, CML is a genetically heterogeneous disease. Many years of research on *BCR*::*ABL1* kinase domain mutations have demonstrated the importance of monitoring TKI resistance and the appropriate TKI selection. TKI therapy has overcome the poor risk previously associated with derivative chromosome 9 deletions and variant translocations. There remains controversy regarding the impact of specific *BCR*::*ABL1* transcript types on treatment outcomes. The advent of large scale and sensitive sequencing technologies has revealed a more genomically complex and heterogeneous disease in select CML patients, especially those with inferior outcomes. This genetic heterogeneity may yield potential future biomarkers of treatment outcomes. The novel discovery of Ph-associated rearrangements already demonstrates a previously unknown genomic mechanism that has the potential to impact patient outcomes. Recent studies of complex rearrangements in other leukemias and solid tumors have also reported an association with poorer outcomes. The co-occurrence of *BCR*::*ABL1* kinase domain mutations and cancer gene mutations in a high proportion of resistant patients indicates that we should not consider resistance as either *BCR*::*ABL1*-dependent or *BCR*::*ABL1*-independent. Rather, resistance mechanisms may act interdependently to drive disease progression. We predict that expanded NGS testing for rearrangements and cancer gene mutations will enhance risk prediction, more reliably classify TKI resistance, and identify novel future targets for therapy.

## Figures and Tables

**Table 1:** *BCR*::*ABL1* kinase domain mutations, which include overlapping myristoyl-binding pocket mutations, that confer resistance to asciminib as predicted in preclinical studies and identified in patients treated with asciminib in a clinical trial.

Study	*BCR::ABL1* Kinase Domain Mutations Detected in Preclinical Studies	*BCR::ABL1* Kinase Domain Mutations Detected in Patient Samples (Hughes et al. [46])
Lee and Shah [51]	A337V	A337T G463D P465S V468F I502L
	P465S	
	V468F	
Wylie et al. [7]	A337V	
	P465S	
	V468F	
	I502L	
Eide et al. [47]	A344P	
	Y353C	
	P465S	

## Data Availability

Not applicable.

## References

[1] Bruford E.A., Antonescu C.R., Carroll A.J., Chinnaiyan A., Cree I.A., Cross N.C.P., Dalgleish R., Gale R.P., Harrison C.J., Hastings R.J. (2021). HUGO Gene Nomenclature Committee (HGNC) recommendations for the designation of gene fusions. Leukemia.

[2] Quintás-Cardama A., Cortes J. (2009). Molecular biology of bcr-abl1–positive chronic myeloid leukemia. Blood.

[3] Hochhaus A., Baccarani M., Silver R.T., Schiffer C., Apperley J.F., Cervantes F., Clark R.E., Cortes J.E., Deininger M.W., Guilhot F. (2020). European LeukemiaNet 2020 recommendations for treating chronic myeloid leukemia. Leukemia.

[4] NCCN Clinical Practice Guidelines in Oncology: Chronic Myeloid Leukemia. Version 2.2022. https://www.nccn.org/guidelines/guidelines-detail?category=1&id=1427.

[5] Wedelin C., Björkholm M., Mellstedt H., Gahrton G., Holm G. (2009). Clinical Findings and Prognostic Factors in Chronic Myeloid Leukemias. Acta Med. Scand..

[6] Daley G.Q., Van Etten R.A., Baltimore D. (1990). Induction of Chronic Myelogenous Leukemia in Mice by the P210 bcr/abl Gene of the Philadelphia Chromosome. Science.

[7] Wylie A.A., Schoepfer J., Jahnke W., Cowan-Jacob S.W., Loo A., Furet P., Marzinzik A.L., Pelle X., Donovan J., Zhu W. (2017). The allosteric inhibitor ABL001 enables dual targeting of BCR–ABL1. Nature.

[8] Jain P., Kantarjian H.M., Ghorab A., Sasaki K., Jabbour E.J., Gonzalez G.N., Kanagal-Shamanna R., Issa G.C., Garcia-Manero G., Kc D. (2017). Prognostic factors and survival outcomes in patients with chronic myeloid leukemia in blast phase in the tyrosine kinase inhibitor era: Cohort study of 477 patients. Cancer.

[9] Hughes T., Ross D. (2016). Moving treatment-free remission into mainstream clinical practice in CML. Blood.

[10] Soverini S., Martinelli G., Rosti G., Bassi S., Amabile M., Poerio A., Giannini B., Trabacchi E., Castagnetti F., Testoni N. (2005). ABL Mutations in Late Chronic Phase Chronic Myeloid Leukemia Patients with Up-Front Cytogenetic Resistance to Imatinib Are Associated with a Greater Likelihood of Progression to Blast Crisis and Shorter Survival: A Study by the GIMEMA Working Party on Chronic Myeloid Leukemia. J. Clin. Oncol..

[11] Branford S., Wang P., Yeung D.T., Thomson D., Purins A., Wadham C., Shahrin N.H., Marum J.E., Nataren N., Parker W.T. (2018). Integrative genomic analysis reveals cancer-associated mutations at diagnosis of CML in patients with high-risk disease. Blood.

[12] Gorre M.E., Mohammed M., Ellwood K., Hsu N., Paquette R., Rao P.N., Sawyers C.L. (2001). Clinical Resistance to STI-571 Cancer Therapy Caused by BCR-ABL Gene Mutation or Amplification. Science.

[13] Shah N.P., Nicoll J.M., Nagar B., Gorre M.E., Paquette R.L., Kuriyan J., Sawyers C.L. (2002). Multiple BCR-ABL kinase domain mutations confer polyclonal resistance to the tyrosine kinase inhibitor imatinib (STI571) in chronic phase and blast crisis chronic myeloid leukemia. Cancer Cell.

[14] Branford S., Rudzki Z., Walsh S., Grigg A., Arthur C., Taylor K., Herrmann R., Lynch K.P., Hughes T.P. (2002). High frequency of point mutations clustered within the adenosine triphosphate–binding region of BCR/ABL in patients with chronic myeloid leukemia or Ph-positive acute lymphoblastic leukemia who develop imatinib (STI571) resistance. Blood.

[15] Von Bubnoff N., Schneller F., Peschel C., Duyster J. (2002). BCR-ABL gene mutations in relation to clinical resistance of Philadelphia-chromosome-positive leukaemia to STI571: A prospective study. Lancet.

[16] Roche-Lestienne C., Soenen V., Grardel N., Laï J.-L., Philippe N., Facon T., Fenaux P., Preudhomme C. (2002). Several types of mutations of the Abl gene can be found in chronic myeloid leukemia patients resistant to STI571, and they can pre-exist to the onset of treatment. Blood.

[17] Branford S., Rudzki Z., Walsh S., Parkinson I., Grigg A., Szer J., Taylor K., Herrmann R., Seymour J.F., Arthur C. (2003). Detection of BCR-ABL mutations in patients with CML treated with imatinib is virtually always accompanied by clinical resistance, and mutations in the ATP phosphate-binding loop (P-loop) are associated with a poor prognosis. Blood.

[18] Jabbour E., Kantarjian H.M., Jones D.T.L., Talpaz M., Bekele N., Obrien S.J., Zhou X., Luthra R., Garciamanero G., Giles F.J. (2006). Frequency and clinical significance of BCR-ABL mutations in patients with chronic myeloid leukemia treated with imatinib mesylate. Leukemia.

[19] Nicolini F., Corm S., Lê Q.-H., Sorel N., Hayette S., Bories D., Leguay T., Roy L., Giraudier S., Tulliez M. (2006). Mutation status and clinical outcome of 89 imatinib mesylate-resistant chronic myelogenous leukemia patients: A retrospective analysis from the French intergroup of CML (Fi(ϕ)-LMC GROUP). Leukemia.

[20] Druker B.J., Tamura S., Buchdunger E., Ohno S., Segal G.M., Fanning S., Zimmermann J., Lydon N.B. (1996). Effects of a selective inhibitor of the Abl tyrosine kinase on the growth of Bcr–Abl positive cells. Nat. Med..

[21] Soverini S., Hochhaus A., Nicolini F.E., Gruber F., Lange T., Saglio G., Pane F., Müller M.C., Ernst T., Rosti G. (2011). BCR-ABL kinase domain mutation analysis in chronic myeloid leukemia patients treated with tyrosine kinase inhibitors: Recommendations from an expert panel on behalf of European LeukemiaNet. Blood.

[22] Thomas E., Philipp E., Martin C.M., Peter P., Thomas S., Jana H., Sebastian K., Paul R., Rüdiger H., Andreas H. (2008). Dynamics of BCR-ABL mutated clones prior to hematologic or cytogenetic resistance to imatinib. Haematologica.

[23] Hughes T., Saglio G., Branford S., Soverini S., Kim D.-W., Müller M.C., Martinelli G., Cortes J., Beppu L., Gottardi E. (2009). Impact of Baseline BCR-ABL Mutations on Response to Nilotinib in Patients with Chronic Myeloid Leukemia in Chronic Phase. J. Clin. Oncol..

[24] Müller M.C., Cortes J., Kim D.-W., Druker B., Erben P., Pasquini R., Branford S., Hughes T., Radich J.P., Ploughman L. (2009). Dasatinib treatment of chronic-phase chronic myeloid leukemia: Analysis of responses according to preexisting BCR-ABL mutations. Blood.

[25] Branford S., Melo J.V., Hughes T.P. (2009). Selecting optimal second-line tyrosine kinase inhibitor therapy for chronic myeloid leukemia patients after imatinib failure: Does the BCR-ABL mutation status really matter?. Blood.

[26] Schindler T., Bornmann W., Pellicena P., Miller W.T., Clarkson B., Kuriyan J. (2000). Structural Mechanism for STI-571 Inhibition of Abelson Tyrosine Kinase. Science.

[27] Nicolini F.E., Mauro M.J., Martinelli G., Kim D.-W., Soverini S., Müller M.C., Hochhaus A., Cortes J., Chuah C., Dufva I.H. (2009). Epidemiologic study on survival of chronic myeloid leukemia and Ph(+) acute lymphoblastic leukemia patients with BCR-ABL T315I mutation. Blood.

[28] Nicolini F.E., Ibrahim A.R., Soverini S., Martinelli G., Müller M.C., Hochhaus A., Dufva I.H., Kim D.-W., Cortes J., Mauro M.J. (2013). The BCR-ABLT315I mutation compromises survival in chronic phase chronic myelogenous leukemia patients resistant to tyrosine kinase inhibitors, in a matched pair analysis. Haematologica.

[29] O’Hare T., Shakespeare W.C., Zhu X., Eide C.A., Rivera V.M., Wang F., Adrian L.T., Zhou T., Huang W.-S., Xu Q. (2009). AP24534, a Pan-BCR-ABL Inhibitor for Chronic Myeloid Leukemia, Potently Inhibits the T315I Mutant and Overcomes Mutation-Based Resistance. Cancer Cell.

[30] Soverini S., Colarossi S., Gnani A., Rosti G., Castagnetti F., Poerio A., Iacobucci I., Amabile M., Abruzzese E., Orlandi E. (2006). Contribution of ABL Kinase Domain Mutations to Imatinib Resistance in Different Subsets of Philadelphia-Positive Patients: By the GIMEMA Working Party on Chronic Myeloid Leukemia. Clin. Cancer Res..

[31] Khorashad J.S., de Lavallade H., Apperley J.F., Milojkovic D., Reid A.G., Bua M., Szydlo R., Olavarria E., Kaeda J., Goldman J.M. (2008). Finding of Kinase Domain Mutations in Patients with Chronic Phase Chronic Myeloid Leukemia Responding to Imatinib May Identify Those at High Risk of Disease Progression. J. Clin. Oncol..

[32] Quintás-Cardama A., Kantarjian H., O’Brien S., Jabbour E., Borthakur G., Ravandi F., Verstovsek S., Shan J., Cortes J. (2011). Outcome of patients with chronic myeloid leukemia with multiple ABL1 kinase domain mutations receiving tyrosine kinase inhibitor therapy. Haematologica.

[33] Kizilors A., Crisà E., Lea N., Passera R., Mian S., Anwar J., Best S., Nicolini F., Ireland R., Aldouri M. (2019). Effect of low-level BCR-ABL1 kinase domain mutations identified by next-generation sequencing in patients with chronic myeloid leukaemia: A population-based study. Lancet Haematol..

[34] Parker W.T., Ho M., Scott H.S., Hughes T.P., Branford S. (2012). Poor response to second-line kinase inhibitors in chronic myeloid leukemia patients with multiple low-level mutations, irrespective of their resistance profile. Blood.

[35] Zabriskie M.S., Eide C.A., Tantravahi S.K., Vellore N.A., Estrada J., Nicolini F.E., Khoury H.J., Larson R., Konopleva M., Cortes J. (2014). BCR-ABL1 Compound Mutations Combining Key Kinase Domain Positions Confer Clinical Resistance to Ponatinib in Ph Chromosome-Positive Leukemia. Cancer Cell.

[36] Parker W.T., Yeung D.T.O., Yeoman A.L., Altamura H.K., Jamison B.A., Field C.R., Hodgson J.G., Lustgarten S., Rivera V.M., Hughes T. (2016). The impact of multiple low-level BCR-ABL1 mutations on response to ponatinib. Blood.

[37] Parker W.T., Lawrence R.M., Ho M., Irwin D.L., Scott H.S., Hughes T.P., Branford S. (2011). Sensitive Detection of BCR-ABL1 Mutations in Patients with Chronic Myeloid Leukemia After Imatinib Resistance Is Predictive of Outcome During Subsequent Therapy. J. Clin. Oncol..

[38] Soverini S., Bavaro L., De Benedittis C., Martelli M., Iurlo A., Orofino N., Sica S., Sorà F., Lunghi F., Ciceri F. (2020). Prospective assessment of NGS-detectable mutations in CML patients with nonoptimal response: The NEXT-in-CML study. Blood.

[39] Soverini S., De Benedittis C., Polakova K.M., Linhartova J., Castagnetti F., Gugliotta G., Papayannidis C., Mancini M., Klamová H., Salvucci M. (2016). Next-generation sequencing for sensitive detection of BCR-ABL1 mutations relevant to tyrosine kinase inhibitor choice in imatinib-resistant patients. Oncotarget.

[40] Polakova K.M., Kulvait V., Benesova A., Linhartova J., Klamova H., Jaruskova M., De Benedittis C., Haferlach T., Baccarani M., Martinelli G. (2014). Next-generation deep sequencing improves detection of BCR-ABL1 kinase domain mutations emerging under tyrosine kinase inhibitor treatment of chronic myeloid leukemia patients in chronic phase. J. Cancer Res. Clin. Oncol..

[41] Erbilgin Y., Eskazan A.E., Ng O.H., Salihoglu A., Elverdi T., Firtina S., Tasar O., Mercan S., Sisko S., Khodzhaev K. (2018). Deep sequencing of BCR-ABL1 kinase domain mutations in chronic myeloid leukemia patients with resistance to tyrosine kinase inhibitors. Leuk. Lymphoma.

[42] Schoepfer J., Jahnke W., Berellini G., Buonamici S., Cotesta S., Cowan-Jacob S.W., Dodd S., Drueckes P., Fabbro D., Gabriel T. (2018). Discovery of Asciminib (ABL001), an Allosteric Inhibitor of the Tyrosine Kinase Activity of BCR-ABL1. J. Med. Chem..

[43] Garcia-Gutiérrez V., Luna A., Alonso-Dominguez J.M., Estrada N., Boque C., Xicoy B., Giraldo P., Angona A., Alvarez-Larrán A., Sanchez-Guijo F. (2021). Safety and efficacy of asciminib treatment in chronic myeloid leukemia patients in real-life clinical practice. Blood Cancer J..

[44] Réa D., Mauro M.J., Boquimpani C., Minami Y., Lomaia E., Voloshin S., Turkina A.G., Kim D.-W., Apperley J.F., Abdo A. (2021). A phase 3, open-label, randomized study of asciminib, a STAMP inhibitor, vs bosutinib in CML after 2 or more prior TKIs. Blood.

[45] Zhang J., Adrián F.J., Jahnke W., Cowan-Jacob S.W., Li A.G., Iacob R.E., Sim T., Powers J., Dierks C., Sun F. (2010). Targeting Bcr–Abl by combining allosteric with ATP-binding-site inhibitors. Nature.

[46] Hughes T.P., Mauro M.J., Cortes J., Minami H., Rea D., DeAngelo D., Breccia M., Goh Y.-T., Talpaz M., Hochhaus A. (2019). Asciminib in Chronic Myeloid Leukemia after ABL Kinase Inhibitor Failure. N. Engl. J. Med..

[47] Eide C.A., Zabriskie M.S., Stevens S.L.S., Antelope O., Vellore N.A., Than H., Schultz A.R., Clair P., Bowler A.D., Pomicter A. (2019). Combining the Allosteric Inhibitor Asciminib with Ponatinib Suppresses Emergence of and Restores Efficacy against Highly Resistant BCR-ABL1 Mutants. Cancer Cell.

[48] Lindström H.J.G., Friedman R. (2020). The effects of combination treatments on drug resistance in chronic myeloid leukaemia: An evaluation of the tyrosine kinase inhibitors axitinib and asciminib. BMC Cancer.

[49] Luskin M.R., Stevenson K.E., Mendez L.M., Wang E.S., Wadleigh M., Garcia J.S., Stone R.M., An H.H., Hagopian E., Galinsky I. (2021). A Phase I Study of Asciminib (ABL001) in Combination with Dasatinib and Prednisone for BCR-ABL1-Positive ALL in Adults. Blood.

[50] Gleixner K.V., Filik Y., Berger D., Schewzik C., Stefanzl G., Sadovnik I., Degenfeld-Schonburg L., Eisenwort G., Schneeweiss-Gleixner M., Byrgazov K. (2021). Asciminib and ponatinib exert synergistic anti-neoplastic effects on CML cells expressing BCR-ABL1 (T315I)-compound mutations. Am. J. Cancer Res..

[51] Lee B.J., Shah N.P. (2016). Identification and characterization of activating ABL1 1b kinase mutations: Impact on sensitivity to ATP-competitive and allosteric ABL1 inhibitors. Leukemia.

[52] Branford S., Hughes T.P., Rudzki Z. (2002). Dual transcription of b2a2 and b3a2 BCR-ABL transcripts in chronic myeloid leukaemia is confined to patients with a linked polymorphism within the BCR gene. Br. J. Haematol..

[53] Baccarani M., Castagnetti F., Gugliotta G., Rosti G., Soverini S., Albeer A., Pfirrmann M. (2019). The proportion of different BCR-ABL1 transcript types in chronic myeloid leukemia. An international overview. Leukemia.

[54] Saglio G., Guerrasio A., Rosso C., Zaccaria A., Tassinari A., Serra A., Cambrin G.R., Mazza U., Gavosto F. (1990). New type of Bcr/Abl junction in Philadelphia chromosome-positive chronic myelogenous leukemia. Blood.

[55] Sharma P., Kumar L., Mohanty S., Kochupillai V. (2009). Response to Imatinib mesylate in chronic myeloid leukemia patients with variant BCR-ABL fusion transcripts. Ann. Hematol..

[56] Molica M., Abruzzese E., Breccia M. (2020). Prognostic Significance of Transcript-Type BCR-ABL1 in Chronic Myeloid Leukemia. Mediterr. J. Hematol. Infect. Dis..

[57] Marum J.E., Branford S. (2016). Current developments in molecular monitoring in chronic myeloid leukemia. Ther. Adv. Hematol..

[58] Shepherd P., Suffolk R., Halsey J., Allan N. (1995). Analysis of molecular breakpoint and m-RNA transcripts in a prospective randomized trial of interferon in chronic myeloid leukaemia: No correlation with clinical features, cytogenetic response, duration of chronic phase, or survival. Br. J. Haematol..

[59] Schaefer-Rego K., Dudek H., Popenoe D., Arlin Z., Mears J., Bank A., Leibowitz D. (1987). CML patients in blast crisis have breakpoints localized to a specific region of the BCR. Blood.

[60] Baccarani M., Tura S., Russo D., Baccarani M., Zaccaria A., Saglio G., Guerrasio A., Martinelli G., Zuffa E., Testoni N. (1995). Chronic myeloid-leukemia, BCR/ABL transcript, response to alpha-interferon and survival. Leukemia.

[61] Hanfstein B., Lauseker M., Hehlmann R., Saussele S., Erben P., Dietz C., Fabarius A., Proetel U., Schnittger S., Haferlach C. (2014). Distinct characteristics of e13a2 versus e14a2 BCR-ABL1 driven chronic myeloid leukemia under first-line therapy with imatinib. Haematologica.

[62] Castagnetti F., Gugliotta G., Breccia M., Iurlo A., Levato L., Albano F., Vigneri P., Abruzzese E., Rossi G., Rupoli S. (2017). The BCR-ABL1 transcript type influences response and outcome in Philadelphia chromosome-positive chronic myeloid leukemia patients treated frontline with imatinib. Am. J. Hematol..

[63] Lucas C., Harris R.J., Giannoudis A., Davies A., Knight K., Watmough S.J., Wang L., Clark R.E. (2009). Chronic myeloid leukemia patients with the e13a2 BCR-ABL fusion transcript have inferior responses to imatinib compared to patients with the e14a2 transcript. Haematologica.

[64] Pagnano K.B.B., Miranda E.C., Delamain M.T., Duarte G.O., de Paula E.V., Lorand-Metze I., de Souza C.A. (2017). Influence of BCR-ABL Transcript Type on Outcome in Patients with Chronic-Phase Chronic Myeloid Leukemia Treated with Imatinib. Clin. Lymphoma Myeloma Leuk..

[65] Polampalli S., Choughule A., Negi N., Shinde S., Baisane C., Amre P., Subramanian P., Gujral S., Prabhash K., Parikh P. (2008). Analysis and comparison of clinicohematological parameters and molecular and cytogenetic response of two Bcr/Abl fusion transcripts. Genet. Mol. Res..

[66] Jain P., Kantarjian H., Patel K.P., Gonzalez G.N., Luthra R., Kanagal-Shamanna R., Sasaki K., Jabbour E., Romo C.G., Kadia T.M. (2016). Impact of BCR-ABL transcript type on outcome in patients with chronic-phase CML treated with tyrosine kinase inhibitors. Blood.

[67] Breccia M., Molica M., Colafigli G., Massaro F., Quattrocchi L., Latagliata R., Mancini M., Diverio D., Guarini A., Alimena G. (2017). Prognostic factors associated with a stable MR4.5 achievement in chronic myeloid leukemia patients treated with imatinib. Oncotarget.

[68] Shanmuganathan N., Pagani I.S., Ross D.M., Park S., Yong A.S.M., Braley J.A., Altamura H.K., Hiwase D.K., Yeung D.T., Kim D.-W. (2021). Early BCR-ABL1 kinetics are predictive of subsequent achievement of treatment-free remission in chronic myeloid leukemia. Blood.

[69] D’Adda M., Farina M., Schieppati F., Borlenghi E., Bottelli C., Cerqui E., Ferrari S., Gramegna D., Pagani C., Passi A. (2019). The e13a2 BCR-ABL transcript negatively affects sustained deep molecular response and the achievement of treatment-free remission in patients with chronic myeloid leukemia who receive tyrosine kinase inhibitors. Cancer.

[70] Claudiani S., Apperley J.F., Gale R.P., Clark R., Szydlo R., Deplano S., Palanicawandar R., Khorashad J., Foroni L., Milojkovic D. (2017). E14a2 BCR-ABL1 transcript is associated with a higher rate of treatment-free remission in individuals with chronic myeloid leukemia after stopping tyrosine kinase inhibitor therapy. Haematologica.

[71] Rojas J.M., Knight K., Wang L., Clark R. (2007). Clinical evaluation of BCR-ABL peptide immunisation in chronic myeloid leukaemia: Results of the EPIC study. Leukemia.

[72] Greiner J., Schmitt M. (2008). Leukemia-associated antigens as target structures for a specific immunotherapy in chronic myeloid leukemia. Eur. J. Haematol..

[73] Dragani M., Petiti J., Rege-Cambrin G., Gottardi E., Daraio F., Caocci G., Aguzzi C., Crisà E., Andreani G., Caciolli F. (2020). Treatment-free remission in Chronic Myeloid Leukemia harboring atypical BCR-ABL1 transcripts. Mediterr. J. Hematol. Infect. Dis..

[74] Pagani I.S., Dang P., Saunders V.A., Braley J., Thieleke A., Branford S., Hughes T.P., Ross D.M. (2020). Clinical utility of genomic DNA Q-PCR for the monitoring of a patient with atypical e19a2 BCR-ABL1 transcripts in chronic myeloid leukemia. Leuk. Lymphoma.

[75] Huret J.L. (1990). Complex translocations, simple variant translocations and Ph-negative cases in chronic myelogenous leukaemia. Qual. Life Res..

[76] Gorusu M., Benn P., Li Z., Fang M. (2007). On the genesis and prognosis of variant translocations in chronic myeloid leukemia. Cancer Genet. Cytogenet..

[77] Marzocchi G., Castagnetti F., Luatti S., Baldazzi C., Stacchini M., Gugliotta G., Amabile M., Specchia G., Sessarego M., Giussani U. (2011). Variant Philadelphia translocations: Molecular-cytogenetic characterization and prognostic influence on frontline imatinib therapy, a GIMEMA Working Party on CML analysis. Blood.

[78] Fitzgerald P.H., Morris C.M. (1991). Complex chromosomal translocations in the Philadelphia chromosome leukemias: Serial translocations or a concerted genomic rearrangement?. Cancer Genet. Cytogenet..

[79] Naumann S., Decker H.-J. (2003). Genesis of variant philadelphia chromosome translocations in chronic myelocytic leukemia. Cancer Genet. Cytogenet..

[80] Calabrese G., Stuppia L., Franchi P.G., Peila R., Morizio E., Liberati A.M., Spadano A., Di Lorenzo R., Donti E., Antonucci A. (1994). Complex translocations of the Ph chromosome and Ph negative CML arise from similar mechanisms, as evidenced by FISH analysis. Cancer Genet. Cytogenet..

[81] Heim S., Billström R., Kristoffersson U., Mandahl N., Strömbeck B., Mitelman F. (1985). Variant Ph translocations in chronic myeloid leukemia. Cancer Genet. Cytogenet..

[82] Sinclair P.B., Nacheva E.P., Leversha M., Telford N., Chang J., Reid A., Bench A., Champion K., Huntly B., Green A.R. (2000). Large deletions at the t(9;22) breakpoint are common and may identify a poor-prognosis subgroup of patients with chronic myeloid leukemia. Blood.

[83] El-Zimaity M.M.T., Kantarjian H., Talpaz M., O’Brien S., Giles F., Garcia-Manero G., Verstovsek S., Thomas D., Ferrajoli A., Hayes K. (2004). Results of imatinib mesylate therapy in chronic myelogenous leukaemia with variant Philadelphia chromosome. Br. J. Haematol..

[84] Huntly B., Reid A.G., Bench A.J., Campbell L.J., Telford N., Shepherd P., Szer J., Prince H.M., Turner P., Grace C. (2001). Deletions of the derivative chromosome 9 occur at the time of the Philadelphia translocation and provide a powerful and independent prognostic indicator in chronic myeloid leukemia. Blood.

[85] Bennour A., Sennana H., Laatiri M.A., Elloumi M., Khelif A., Saad A. (2009). Molecular cytogenetic characterization of variant Philadelphia translocations in chronic myeloid leukemia: Genesis and deletion of derivative chromosome 9. Cancer Genet. Cytogenet..

[86] Reid A.G., Huntly B., Grace C., Green A., Nacheva E.P. (2003). Survival implications of molecular heterogeneity in variant Philadelphia-positive chronic myeloid leukaemia. Br. J. Haematol..

[87] Potter A.M., Watmore A.E., Cooke P., Lilleyman J.S., Sokol R.J. (1981). Significance of non-standard Philadelphia chromosomes in chronic granulocytic leukaemia. Br. J. Cancer.

[88] Kanakasetty G.B., Kuntejowdahalli L., Thanky A.H., Dasappa L., Jacob L.A., Mallekavu S.B., Kumari P. (2016). Predictive and Prognostic Implications of Variant Philadelphia Translocations in CML: Experience from a Tertiary Oncology Center in Southern India. Clin. Lymphoma Myeloma Leuk..

[89] Koshiyama D.B., Capra M.E.Z., Paskulin G.A., Rosa R.F.M., Oliveira C.A.V., Vanelli T., Fogliatto L.M., Zen P.R.G. (2012). Cytogenetic response to imatinib treatment in Southern Brazilian patients with chronic myelogenous leukemia and variant Philadelphia chromosome. Ann. Hematol..

[90] Fabarius A., Leitner A., Hochhaus A., Müller M.C., Hanfstein B., Haferlach C., Göhring G., Schlegelberger B., Jotterand M., Reiter A. (2011). Impact of additional cytogenetic aberrations at diagnosis on prognosis of CML: Long-term observation of 1151 patients from the randomized CML Study IV. Blood.

[91] Eyüpoğlu D., Bozkurt S., Haznedaroğlu I., Büyükaşık Y., Güven D. (2016). The Impact of Variant Philadelphia Chromosome Translocations on the Clinical Course of Chronic Myeloid Leukemia. Turk. J. Haematol..

[92] Richebourg S., Eclache V., Perot C., Portnoi M.-F., Akker J.V.D., Terre C., Maareck O., Soenen V., Viguié F., Lai J.-L. (2008). Mechanisms of genesis of variant translocation in chronic myeloid leukemia are not correlated with ABL1 or BCR deletion status or response to imatinib therapy. Cancer Genet. Cytogenet..

[93] Stagno F., Vigneri P., Del Fabro V., Stella S., Cupri A., Massimino M., Consoli C., Tambè L., Consoli M.L., Antolino A. (2010). Influence of complex variant chromosomal translocations in chronic myeloid leukemia patients treated with tyrosine kinase inhibitors. Acta Oncol..

[94] Al-Achkar W., Liehr T., Wafa A. (2010). Insertion of the 3′ ABL region into the long arm of chromosome 1 in a Philadelphia chromosome-negative chronic myeloid leukemia case. Oncol. Lett..

[95] Seong D., Kantarjian H., Albitar M., Arlinghaus R., Xu J., Talpaz M., Rios M.B., Guo J.Q., O’Brien S., Siciliano M. (1999). Analysis of Philadelphia chromosome-negative BCR-ABL-positive chronic myelogenous leukemia by hypermetaphase fluorescence in situ hybridization. Ann. Oncol..

[96] Fitzgerald P.H., Beard M.E.J., Morris C.M., Heaton D.C., Reeve A.E. (1987). Ph-negative chronic myeloid leukaemia. Br. J. Haematol..

[97] Van Der Plas D., Hermans A., Soekarman D., Smit E., De Klein A., Smadja N., Alimena G., Goudsmit R., Grosveld G., Hagemeijer A. (1989). Cytogenetic and molecular analysis in Philadelphia negative CML. Blood.

[98] Virgili A., Brazma D., Reid A.G., Howard-Reeves J., Valgañón M., Chanalaris A., De Melo V.A., Marin D., Apperley J.F., Grace C. (2008). FISH mapping of Philadelphia negative BCR/ABL1 positive CML. Mol. Cytogenet..

[99] Baccarani M., Deininger M.W., Rosti G., Hochhaus A., Soverini S., Apperley J.F., Cervantes F., Clark R.E., Cortes J.E., Guilhot F. (2013). European LeukemiaNet recommendations for the management of chronic myeloid leukemia: 2013. Blood.

[100] Huntly B.J.P., Bench A., Green A.R. (2003). Double jeopardy from a single translocation: Deletions of the derivative chromosome 9 in chronic myeloid leukemia. Blood.

[101] Quintás-Cardama A., Kantarjian H., Shan J., Jabbour E., Abruzzo L.V., Verstovsek S., Garcia-Manero G., O’Brien S., Cortes J. (2011). Prognostic impact of deletions of derivative chromosome 9 in patients with chronic myelogenous leukemia treated with nilotinib or dasatinib. Cancer.

[102] Zhang H., Liu M., Wang X., Ren Y., Kim Y.M., Wang X., Lu X., Pang H., Liu G., Gu Y. (2021). Genomic Copy Number Variants in CML Patients with the Philadelphia Chromosome (Ph+): An Update. Front. Genet..

[103] Kolomietz E., Al-Maghrabi J., Brennan S., Karaskova J., Minkin S., Lipton J., Squire J.A. (2001). Primary chromosomal rearrangements of leukemia are frequently accompanied by extensive submicroscopic deletions and may lead to altered prognosis. Blood.

[104] Quintas-Cardama A., Kantarjian H., Talpaz M., O’Brien S., Garcia-Manero G., Verstovsek S., Rios M.B., Hayes K., Glassman A., Bekele B.N. (2005). Imatinib mesylate therapy may overcome the poor prognostic significance of deletions of derivative chromosome 9 in patients with chronic myelogenous leukemia. Blood.

[105] Castagnetti F., Testoni N., Luatti S., Marzocchi G., Mancini M., Kerim S., Giugliano E., Albano F., Cuneo A., Abruzzese E. (2010). Deletions of the Derivative Chromosome 9 Do Not Influence the Response and the Outcome of Chronic Myeloid Leukemia in Early Chronic Phase Treated with Imatinib Mesylate: GIMEMA CML Working Party Analysis. J. Clin. Oncol..

[106] Baccarani M., Saglio G., Goldman J., Hochhaus A., Simonsson B., Appelbaum F., Apperley J., Cervantes F., Cortes J., Deininger M. (2006). Evolving concepts in the management of chronic myeloid leukemia: Recommendations from an expert panel on behalf of the European LeukemiaNet. Blood.

[107] Švabek T., Josipović M., Horvat I., Zadro R., Davidović-Mrsić S. (2018). The incidence of atypical patterns ofBCR-ABL1rearrangement and molecular-cytogenetic response to tyrosine kinase inhibitor therapy in newly diagnosed cases with chronic myeloid leukemia (CML). Blood Res..

[108] Bacher U., Schnittger S., Kern W., Hiddemann W., Haferlach T., Schoch C. (2005). The incidence of submicroscopic deletions in reciprocal translocations is similar in acute myeloid leukemia, BCR-ABL positive acute lymphoblastic leukemia, and chronic myeloid leukemia. Haematologica.

[109] Moon H.W., Chang Y.H., Kim T.Y., Oh B.R., Min H.C., Kim B.K., Ahn H.S., Cho H.I., Lee D.S. (2007). Incidence of submicroscopic deletions vary according to disease entities and chromosomal translocations in hematologic malignancies: Investigation by fluorescence in situ hybridization. Cancer Genet. Cytogenet..

[110] Kim J.C., Zuzarte P.C., Murphy T., Chan-Seng-Yue M., Brown A.M.K., Krzyzanowski P.M., Smith A.C., Notta F., Minden M.D., McPherson J.D. (2019). Cryptic genomic lesions in adverse-risk acute myeloid leukemia identified by integrated whole genome and transcriptome sequencing. Leukemia.

[111] Ma E., Wan T.S., Au C.H., Ho D.N., Ma S.Y., Ng M.H., Chan T.L. (2017). Next-generation sequencing and molecular cytogenetic characterization of ETV6-LYN fusion due to chromosomes 1, 8 and 12 rearrangement in acute myeloid leukemia. Cancer Genet..

[112] Ishii Y., Sashida G., Takaku T.-I., Sumi M., Nakajima A., Ohyashiki K. (2005). Cryptic chromosomal anomaly in a patient with acute myeloid leukemia leading to AML1/ETO fusion with unfavorable prognostic factors. Cancer Genet. Cytogenet..

[113] Andersson A.K., Ma J., Wang J., Chen X., Gedman A.L., Dang J., Nakitandwe J., Holmfeldt L., Parker M., Easton J. (2015). The landscape of somatic mutations in infant MLL-rearranged acute lymphoblastic leukemias. Nat. Genet..

[114] Yang L., Ding L., Liang J., Tang Y., Xue H., Gu L., Shen S., Li B., Chen J. (2018). Relatively favorable prognosis for MLL -rearranged childhood acute leukemia with reciprocal translocations. Pediatr. Blood Cancer.

[115] Anderson N.D., de Borja R., Young M.D., Fuligni F., Rosic A., Roberts N.D., Hajjar S., Layeghifard M., Novokmet A., Kowalski P.E. (2018). Rearrangement bursts generate canonical gene fusions in bone and soft tissue tumors. Science.

[116] Shen M.M. (2013). Chromoplexy: A New Category of Complex Rearrangements in the Cancer Genome. Cancer Cell.

[117] Baca S.C., Prandi D., Lawrence M.S., Mosquera J.M., Romanel A., Drier Y., Park K., Kitabayashi N., Macdonald T.Y., Ghandi M. (2013). Punctuated Evolution of Prostate Cancer Genomes. Cell.

[118] Kim T., Tyndel M.S., Kim H.J., Ahn J.-S., Choi S.H., Park H.J., Kim Y.-K., Kim S.Y., Lipton J.H., Zhang Z. (2017). Spectrum of somatic mutation dynamics in chronic myeloid leukemia following tyrosine kinase inhibitor therapy. Blood.

[119] Awad S.A., Kankainen M., Ojala T., Koskenvesa P., Eldfors S., Ghimire B., Kumar A., Kytölä S., Kamel M.M., Heckman C.A. (2020). Mutation accumulation in cancer genes relates to nonoptimal outcome in chronic myeloid leukemia. Blood Adv..

[120] Wu W., Xu N., Zhou X., Liu L., Tan Y., Luo J., Huang J., Qin J., Wang J., Li Z. (2020). Integrative Genomic Analysis Reveals Cancer-Associated Gene Mutations in Chronic Myeloid Leukemia Patients with Resistance or Intolerance to Tyrosine Kinase Inhibitor. OncoTargets Ther..

[121] Ko T.K., Javed A., Lee K.L., Pathiraja T.N., Liu X., Malik S., Soh S.X., Heng X.T., Takahashi N., Tan J.H.J. (2020). An integrative model of pathway convergence in genetically heterogeneous blast crisis chronic myeloid leukemia. Blood.

[122] Magistroni V., Mauri M., D’Aliberti D., Mezzatesta C., Crespiatico I., Nava M., Fontana D., Sharma N., Parker W., Schreiber A. (2019). De novo UBE2A mutations are recurrently acquired during chronic myeloid leukemia progression and interfere with myeloid differentiation pathways. Haematologica.

[123] Mangan J.K., Speck N.A. (2011). RUNX1 Mutations in Clonal Myeloid Disorders: From Conventional Cytogenetics to Next Generation Sequencing, A Story 40 Years in the Making. Crit. Rev. Oncog..

[124] Osato M., Asou N., Abdalla E., Hoshino K., Yamasaki H., Okubo T., Suzushima H., Takatsuki K., Kanno T., Shigesada K. (1999). Biallelic and Heterozygous Point Mutations in the Runt Domain of the AML1/PEBP2α B Gene Associated with Myeloblastic Leukemias. Blood J. Am. Soc. Hematol..

[125] Grossmann V., Kohlmann A., Zenger M., Schindela S., Eder C., Weissmann S., Schnittger S., Kern W., Muller M.C., Hochhaus A. (2011). A deep-sequencing study of chronic myeloid leukemia patients in blast crisis (BC-CML) detects mutations in 76.9% of cases. Leukemia.

[126] Ochi Y., Yoshida K., Huang Y.-J., Kuo M.-C., Nannya Y., Sasaki K., Mitani K., Hosoya N., Hiramoto N., Ishikawa T. (2021). Clonal evolution and clinical implications of genetic abnormalities in blastic transformation of chronic myeloid leukaemia. Nat. Commun..

[127] Awad S.A., Dufva O., Ianevski A., Ghimire B., Koski J., Maliniemi P., Thomson D., Schreiber A., Heckman C.A., Koskenvesa P. (2020). RUNX1 mutations in blast-phase chronic myeloid leukemia associate with distinct phenotypes, transcriptional profiles, and drug responses. Leukemia.

[128] Roche-Lestienne C., Marceau A., Labis E., Nibourel O., Coiteux V., Guilhot J., Legros L., Nicolini F., Rousselot P., Gardembas M. (2011). Mutation analysis of TET2, IDH1, IDH2 and ASXL1 in chronic myeloid leukemia. Leukemia.

[129] Adnan-Awad S., Kankainen M., Mustjoki S. (2021). Mutational landscape of chronic myeloid leukemia: More than a single oncogene leukemia. Leuk. Lymphoma.

[130] Branford S., Kim D.D.H., Apperley J.F., Eide C.A., Mustjoki S., Ong S.T., Nteliopoulos G., Ernst T., Chuah C., Gambacorti-Passerini C. (2019). Laying the foundation for genomically-based risk assessment in chronic myeloid leukemia. Leukemia.

[131] Zhang X., Li Z., Qin Y., Gale R.P., Huang X., Jiang Q. (2021). Correlations between Mutations in Cancer-Related Genes, Therapy Responses and Outcomes of the 3 rd Generation Tyrosine Kinase-Inhibitor (TKI) in Persons with Chronic Myeloid Leukemia Failing Prior TKI-Therapy. Blood.

[132] Xue M., Zeng Z., Wang Q., Wen L., Xu Y., Xie J., Wang Q., Ruan C., Wu D., Chen S. (2021). Mutational Profiles during the Progression of Chronic Myeloid Leukemia. Blood.

[133] Thomson D.W., Shahrin N.H., Wang P.P.S., Wadham C., Shanmuganathan N., Scott H.S., Dinger M.E., Hughes T.P., Schreiber A., Branford S. (2020). Aberrant RAG-mediated recombination contributes to multiple structural rearrangements in lymphoid blast crisis of chronic myeloid leukemia. Leukemia.

